# Supplementary material for: Domestic dogs in indigenous Amazonian communities: key players in Leptospira cycling and transmission?
Source: bioRxiv. 2023 Sep 20:2023.09.19.558554. Preprint. [Version 1] doi: 10.1101/2023.09.19.558554 (PMC10541607; doi:10.1101/2023.09.19.558554)
Supplement: Supplement 1 [file NIHPP2023.09.19.558554v1-supplement-1.pdf]

S1 Table. Serological analysis of 48 dogs by MAT

Individual	Community	MAT	Serovars																
			AND	AUS	AUT	BAT	BRA	CAN	DJA	GRI	HAR	HEB	POM	PYR	SAX	SER	SHE	TAR	WOL
M01	Pompeya	N																	
M03	Pompeya	P																100	
M06	Pompeya	N																	
M07	Pompeya	N																	
M09	Pompeya	P															100	100	
M11	San Roque	P		100			100	100		100				100					
M12	San Roque	P		100										100					
M13	San Roque	P		100			100			100	400					100			100
M14	San Roque	P		400				100			100								
M15	San Roque	P														100	100	200	
M16	San Roque	P		400														200	
M17	San Roque	P																100	
M18	San Roque	N																	
M19	San Roque	P												100					
M20	San Roque	P													100				
M21	San Roque	P						100									100		
M22	San Roque	P						100		100					100	100	100	100	100
M23	Sani Isla	P													100	100	100	100	400
M24	Sani Isla	N																	
M25	Sani Isla	P																	100
M26	Sani Isla	P								100									
M27	Sani Isla	N																	
M28	Sani Isla	P						200		100									
M29	Sani Isla	N																	
M30	Sani Isla	N																	
M31	Sani Isla	P							100	100									
M32	Sani Isla	P									100				100				100
M33	Sani Isla	N																	
M34	Sani Isla	P													100				

M35	Nueva Providencia	P														100			
M36	Nueva Providencia	P		100				100											
M37	Nueva Providencia	P												100					
M38	Nueva Providencia	P							100			100		100					
M39	Nueva Providencia	P												100					
M40	Nueva Providencia	N																	
M41	Nueva Providencia	P		100					200						200		100	100	
M42	Nueva Providencia	P															100	100	
M43	Nueva Providencia	N																	
M44	Nueva Providencia	P		100		100					100				100	100		200	100
M45	Nueva Providencia	P														100			100
M46	Nueva Providencia	P		100															
M47	Indillama	N																	
M48	Indillama	P		100														200	
M49	Indillama	P	100					100					100	100				100	100
M50	Indillama	P	100	200		100		200		100	100							100	
M51	Indillama	P						100											
M52	Pompeya	P		100	100	100	100	200	100	200	200					100		100	
M53	Pompeya	P				100											200		

N= negative; P= positive. MAT titers against more than one serovar were detected in 15/36 MAT-positive dogs.

AND= Andamana; AUS=Australis; AUT=Autumnalis; BAT=Bataviae; BRA= Bratislava; CAN=Canicola; DJA=Djasiman; GRI=Grippotyphosa; HAR= Hardjo; HEB=Hebdomadis; POM=Pomona; PYR=Pyrogenes; SAX= Saxkoebing; SEJ=Sejroe; SHE=Shermani; TAR=Tarassovi; WOL= Wolffi.

bioRxiv preprint doi: https://doi.org/10.1101/2023.09.19.558554; this version posted September 20, 2023. The copyright holder for this preprint (which was not certified by peer review) is the author/funder, who has granted bioRxiv a license to display the preprint in perpetuity. It is made available under aCC-BY 4.0 International license.
